# Perinatal continuity of care for mothers with depressive symptoms: perspectives of mothers and clinicians

**DOI:** 10.3389/fpsyt.2024.1385120

**Published:** 2024-09-19

**Authors:** Karlen R. Barr, Trisha A. Nguyen, Wendy Pickup, Sara Cibralic, Antonio Mendoza Diaz, Bryanne Barnett, Valsamma Eapen

**Affiliations:** ^1^ Academic Unit of Infant, Child and Adolescent Psychiatry, South Western Sydney Local Health District, Sydney, NSW, Australia; ^2^ Faculty of Medicine and Health, Discipline of Psychiatry and Mental Health, University of New South Wales, Sydney, NSW, Australia; ^3^ Ingham Institute for Applied Medical Research, Liverpool, NSW, Australia; ^4^ Tasmanian Centre for Mental Health Service Innovation, Tasmanian Health Service, Hobart, TAS, Australia

**Keywords:** continuity of care, perinatal, antenatal, midwife, pregnancy, depression

## Abstract

**Background:**

Mothers with mild to moderate depression in pregnancy are at risk of developing postpartum depression. Midwife-led continuity of care may support maternal mental health throughout the perinatal period. Research is needed to better understand how continuity of care may support mothers experiencing depression in pregnancy. This study aimed to investigate the perspectives of mothers with mild to moderate depression and clinicians regarding continuity of care in the perinatal period.

**Method:**

Fourteen mothers and clinicians participated in individual interviews or a focus group. Analysis was conducted using inductive reflexive thematic analysis with a constructivist orientation.

**Results:**

From the perspectives of mothers and clinicians, continuity of care during the antenatal period benefitted mothers’ mental health by providing connection and rapport, information about pregnancy and referral options, and reassurance about whether pregnancy symptoms were normal. The experience of seeing multiple clinicians was noted by mothers to increase distress while participants discussed the value of extending continuity of care into the postpartum period, including having someone familiar checking in on them. The importance of having a second opinion and not always relying on a single provider during pregnancy was highlighted by some mothers and clinicians. Mothers also described how multiple modes of communication with a midwife can be helpful, including the ease and accessibility of text or email.

**Conclusion:**

Mothers and clinicians perceived benefits of continuity of care for maternal mental health. Offering midwife-led continuity of care to mothers with mild to moderate depression during the perinatal period is recommended.

## 1 Introduction

During the perinatal period, mothers are at increased risk of mental ill-health (1). Undetected mental health difficulties, including depression and anxiety, can negatively impact the mother, their baby and mother-child interactions (2–4). Perinatal mental health conditions are the most common complication of childbearing, associated with considerable negative maternal and birth outcomes (4).

Perinatal mental health screening is a critical tool used to identify at-risk mothers who could benefit from increased support and formal mental health assessment (5). As such, routine screening of mental health problems in pregnant women has been mandated across public maternity services in New South Wales, Australia (6). Despite the benefits associated with screening, the program has been critiqued as the original wide focus of this program was reduced (because of the potential burden on available services) to only those women experiencing high risk and hence women may be falling through the cracks, not receive adequate intervention if they do not meet the high risk threshold (7, 8). Risk for depression in the perinatal period is most commonly screened using the Edinburgh Postnatal Depression Scale (EPDS) (9). Mothers with mild-to-moderate depression with an EPDS score of 10 to 12 have 4.5 times higher odds of experiencing postpartum depression compared with mothers who score 9 or below (9, 10). Nevertheless, women who score 10 to 12 may not meet the threshold to receive referral to appropriate support services. This can be problematic as they experience significant distress, described as having anxiety, mild-to-moderate depression or low psychological resources. It is critical that these women are proactively supported to prevent deterioration of their mental health (10).

Regarding adverse perinatal outcomes, inequity is noted as a key factor (11). Women from culturally and linguistically diverse (CALD) backgrounds may be particularly at risk of perinatal mental health problems (12). Providing midwife-led continuity of care to women throughout pregnancy and the postpartum period may assist the wellbeing of mothers at risk of perinatal mental health problems (13). The key element in midwifery continuity of care is the presence of a known midwife from the initial antenatal appointment through to the postnatal period (14). In addition to relational continuity, midwifery continuity of care also encompasses informational continuity, ensuring that all relevant information pertaining to the woman’s health is shared across settings, and management continuity, providing a coherent and coordinated care pathway (15). In contrast, the shared model of care includes care provided by different midwives, obstetricians and general practitioners (16). The obstetricians and general practitioners who provide antenatal care are often not present for the birth and do not provide postpartum support (14). According to a systematic review and meta-analysis, the physical benefits for women receiving midwife-led continuity of care include fewer episiotomies, epidurals and instrumental births, increased chances of spontaneous vaginal birth, and lower preterm births and stillbirths (14). In addition to physical health benefits, a recent systematic review also found preliminary evidence showing midwifery continuity of care is beneficial in reducing anxiety/worry and depression in pregnant women during the antenatal period (13). However, there has been problems with wide-spread implementation, with limited access to midwifery-led continuity of care in Australia despite a desire to increase this maternity service (17). Currently, the majority of Australian women do not have access to continuity of care across their pregnancy, birth and postpartum period with only around 29% of women having continuity of care throughout the perinatal period (18). This can be attributed to a number of systemic issues. For example, restrictive criteria of having a low-risk pregnancy and the exclusion of women with perinatal mental health conditions from this care model due to psychosocial risk factors (19, 20). Other barriers, such as burnout, a cultural preference towards shift-work, and lack of permanent staff restricts, the provision of continuous care (21–23).

Nevertheless, continuity of care may be beneficial for women with perinatal mental health problems. According to a recent systematic review, there is evidence to suggest that midwife-led continuity of care may lead to reduced perinatal depression and anxiety (13). This may be due to the trusting relationship and shared decision making that women experience through repeated interactions with the same midwife or midwife group (24, 25). Evidence also shows that women with perinatal mental health problems who receive midwife-led continuity of care have lower odds of preterm birth, a risk factor for pregnant women with mental health difficulties (19, 26).

In qualitative research, mothers have emphasised how midwives providing continuity of care aids in help seeking for mental health problems, including increased comfort in disclosing perinatal mental health concerns due to connection and rapport (27–29). Conversely, providers having insufficient time and fragmented models of care were described as barriers to disclosing perinatal mental health problems (27). According to midwives, continuity of care assists in building trusting relationships with women and thereby enabling screening and early identification of the onset of mental health problems (29, 30). There is only a small number of studies on continuity of care for women with mental health problems during the perinatal period, and more research is needed to understand how continuity of care can support these mothers (13). For instance, it is not clear which aspects of continuity of care contribute to improved maternal mental health.

The aim of this study was to understand the perspectives of mothers and clinicians regarding continuity of care for women with mild to moderate depression during the perinatal period.

## 2 Materials and methods

### 2.1 Study context

This study was part of a pilot randomised control trial conducted across two public hospitals in New South Wales, Australia. The study took place within a CALD community in South Western Sydney, New South Wales, with approximately 80% of households primarily communicating in a language other than English (31). Mothers were randomly assigned to receive ongoing care from the same midwife throughout the perinatal period (the intervention group) or received treatment as usual where care was provided by multiple service providers (the control group). Mothers receiving continuity of care could communicate with the same midwife throughout the perinatal period, often by phone or text during the 2021 COVID-19 restrictions. The “usual care” involved women receiving maternity care from a variety of services including midwives, GPs, and private clinics, where participants were seen by any of the staff available at the service. Following recruitment, mothers were followed up at three points in time namely 36 weeks gestation, 8 weeks postpartum and 6 months postpartum to evaluate maternal health, health indicators, connectedness to local services and their perceived quality of service provision. Qualitative interviews were conducted at the 6-month follow-up.

### 2.2 Participants

Eligible mothers were invited to participate in the randomised control trial by the research midwife when they attended their booking visit at the antenatal clinic at around 12 weeks gestation. As part of routine care, when women contacted the clinic reception initially, they were asked to provide personal details alongside other health indicators. Mothers were screened using the EPDS, a questionnaire used to identify women with mental health issues who may benefit from follow-up care, at the booking visit, at 36 weeks gestation, 8 weeks postpartum and 6 months postpartum. Women scoring 10 to 12 on the EPDS (i.e. raised but subclinical threshold for anxiety and depression) at the booking visit were asked whether they would like to participate in the study by the antenatal clinic booking midwife. Contact details of women who had indicated interest were passed onto the research midwife. The clinics were also screened daily for eligible women and contacted by the research midwife. Women were excluded from the study if they had planned on giving birth at a different hospital. In total, 37 participants were recruited to the piolet randomised control trial, with 18 mothers in the intervention group and 19 mothers in the control group. Women from both the intervention and control group as well as staff from the services were approached about participating in the qualitative interviews and interviews were continued until data saturation was reached. Though not excluded from the wider study, women were excluded from the interview if they were unable to speak English proficiently. The final interview process included 14 participants comprising 9 mothers and 5 clinicians. Clinicians included 4 midwives who worked at the antenatal clinic and 1 obstetrician and gynaecologist. Of the 9 mothers, 5 were in the intervention group and 4 were in usual care. Demographic information can be found in **Table 1**.

**Table 1:** Demographic information of participants.

Characteristic	
Mothers (n = 9)	
**Age *M (SD)*, Range**	28.4 (5.9), 21 - 38
**Country of Birth *n* (%)**	
Australia	4 (44%)
Bangladesh	1 (11%)
Iraq	1 (11%)
New Zealand	1 (11%)
Vietnam	2 (22%)
**Care Model *n (%)***	
Doctor Clinic	3 (33%)
Midwife Clinic	5 (56%)
Obstetrician and Gynaecologist	1 (11%)
Clinicians (n = 5)	
**Professional Role *n* (%)**	
Midwife	4 (80%)
Doctor – Obstetrics and Gynaecology training registrar	1 (20%)

### 2.3 Procedure

Ethics approval was obtained for this study (2020/ETH02780) and participants gave written consent prior to the interview. One-on-one interviews were conducted with the majority of participants via telephone. Due to time constraints, a focus group was conducted with three midwives who worked in the same clinic. Interviews followed a semi-structured interview guide that was created by the authors, as outlined in **Appendix 1**. Twelve questions were asked and differed slightly for mothers and clinicians. Questions for mothers explored what it felt like to have access to a midwife, whether having access to the midwife affected their mental health, whether the program was helpful or unhelpful, and whether they had any recommendations (e.g., “What did it feel like to have access to the midwife?”). Clinicians were asked whether they thought that having access to the program would benefit mothers, and any benefits and risks they thought would be related to having a midwife in contact with clients throughout the perinatal period. Interviews were approximately 15 to 30 minutes in length, and took place between August 2022 and April 2023. Most interviews were conducted by female research officers/assistants, one interview was conducted by a male research officer. Transcripts were audio recorded using Zoom and transcribed verbatim. Transcripts were then checked against the recordings by a research assistant to ensure they were accurately transcribed.

### 2.4 Data analysis

Due to the share insights across individual and focus group interviews the data was combined and all interview and focus group data were analysed together. Data was analysed through an inductive approach using reflexive thematic analysis (32, 33). A constructivist orientation was used to investigate the perceptions and experiences of participants by exploring their beliefs and opinions (34, 35). The individual perspectives of participants and the meaning behind their responses were considered when developing the themes. Data analysis was performed by two researchers (KRB and TAN) who individually analysed all transcripts to facilitate a reflexive process (36). One researcher was a clinical psychologist with extensive experience in qualitative analysis, and the other researcher was trained in qualitative methods prior to analysis. First, the researchers familiarised themselves with the data by re-reading the transcripts and listening to the recordings. Data with similar meanings were then coded into nodes using NVivo 12 (37). Codes were refined and candidate themes were created. Candidate themes were revised through discussions with the research team until a final set of themes was established.

## 3 Results

Four major themes were identified.

### 3.1 Theme 1: Merits of the continuity of care model

Many participants shared how continuity of care positively influenced mothers’ mental and physical health through the perinatal period. Three subthemes were developed under this theme regarding improvements in rapport, midwives providing information, and midwives providing reassurance, as described in more detail below.

#### 3.1.1 Subtheme 1.1: Continuity of care may improve rapport with mothers, increase trust and foster effective care

Many participants described how continuity of care throughout the perinatal period would increase trust, connection and rapport between mothers and midwives.

*“When you have that one person seeing them, looking after them, it would just build like a sense of trust”* (Mother 6).

Participants discussed how continued visits with the same midwife helps mothers feel known and relieves them of sharing their story repeatedly with multiple providers.

*“You’ve already built a relationship with them, and you don’t have to go through your history over and over again”* (Mother 3).

Participants also discussed the importance of having a midwife check in on them, particularly when they were experiencing mental health challenges.

“*I just felt like my world is coming to an end, and I was so emotional and to have [the midwife] check in and just say hey, just checking in … that could help another woman that’s probably doing it tough”* (Mother 2).

Additionally, mothers commented how the completion of surveys during the research study allowed them to reflect on their mental health and in turn, be aware if they needed to seek further support.

“*I didn’t realise at some point I was quite low in how I was feeling and by doing the survey, I was able to be aware … [The midwife] even called and checked in on me and I thought that was really personable*” (Mother 7).

According to participants, seeing the same midwife increased rapport and helped mothers open up to clinicians, including sharing their mental health difficulties.

*“I think it certainly is beneficial for mental health because mental health is something that people are often not comfortable discussing until they developed that rapport. So having continuity means there’d be much more likely to disclose things”* (Doctor).

“As they know the midwife, each visit they will be happy to come because they know who is their caregiver, they are familiar … and the chance of them coming to the visit will be higher than someone who is concerned about their visit” (Midwife 1). Participants noted that increased rapport and their comfort in disclosure could improve care provided to these mothers.

*“They’re all a lot more comfortable with just being us during their pregnancy and are happier with the outcome, even if they have a traumatic birth”* (Midwife Focus Group).

One clinician reported that mothers were more likely to disclose mental health concerns and domestic violence issues to their midwives in their native language either through an interpreter or the midwives who can speak the language themselves.

*“Being able to speak in this native language … then the women who have high EPDS who disclosed any issues like domestic violence to their midwives. They tell them more”* (Midwife Focus Group).

All participating mothers stated they would recommend midwife-led continuity of care to a friend.

Some mothers described their experiences of being on a shared care plan and their dislike of seeing different clinicians.

*“It would have been nice to have someone to turn to because when you’re in the public health care system on a shared care plan, you see different people every week in the hospital … I didn’t like that”* (Mother 3).

One mother reflected on the shared care plan with her previous pregnancy and felt, like other participants on the shared care plan, that she was ignored and not provided with personable care.

*“Briefly during my shared care plan in my previous pregnancy, [the doctor] pretty much just ignored my questions and it was just in and out”* (Mother 3).

Some mothers found that having a different clinician each visit caused distress as they were repeatedly questioned and had to reiterate their history.

*“Doctors asked me the same question every single time. Doctors that didn’t know what was going on … Different people, every single appointment … that’s giving me anxiety”* (Mother 6).

*“It felt more pressure having to see the doctor and it was a different doctor each time … The reintroduction of what my history was, what was going on, it was harder” (*Mother 7).

As such, mothers expressed a preference to see a single (continuity of care) midwife.

*“I wish we had the midwife properly in the hospital, at every single appointment and antenatal visit”* (Mother 6).

#### 3.1.2 Subtheme 1.2: Provision of information by the same midwife may support hospital resources

Participants described the value of midwives providing mothers with information during pregnancy.

*“I had other pregnant friends over. I’d say yeah the midwife told me this because they didn’t know where they could go for information”* (Mother 1).

One mother described how the midwife provided information regarding additional supports and referral options.

*“[Access to the same midwife] allowed me to seek out help or additional just support which I normally wouldn’t … It allowed that space and there were resources available and things to be referred to me”* (Mother 7).

Both mothers and clinicians discussed the longer waiting times in the clinic, especially when seeing a doctor.

*“The wait time was much longer for a doctor … with the midwife’s clinic it was just in and out very easy”* (Mother 7).

Due to the information that the same midwife was able to provide to mothers, participants noted that hospital resources were used more efficiently, including a reduction of unnecessary hospital presentations.

*“I didn’t have to call the hospital often that’s for sure”* (Mother 1).

*“[Continuity of care] might even improve the use of our resources. Maybe even prevent some unnecessary presentations to the birthing unit or and things like that because [mothers are] able to access someone that they trust and get some advice about something that they’re experiencing”* (Doctor).

It was noted that mothers in continuity of care “tend to retain that [the midwives] told them and information given to them, beneficial for when they’re in labour” (Midwife Focus Group), which aids in health literacy and use of health services.

#### 3.1.3 Subtheme 1.3: Midwives can provide reassurance when mothers are stressed or have questions

Participants described how mothers could be reassured by their continuity of care midwife, particularly when they are concerned about pregnancy symptoms or have questions, including questions about mental health concerns.

*“You can ask [the midwife] anything that you’re unsure of, yeah like anything you’re not happy about, you can ask them. And they’ll tell you that’s normal that’s not normal”* (Mother 4).

*“When I was pregnant, I was so hormonal. So moody. And I expressed these concerns, and I’m trying to convince myself it’s just because I’m pregnant. And the reassurance of yes don’t stress. It’s normal to feel down”* (Mother 2).

The midwives were also aware of the impact that continuity of care can have on reassuring mothers that they are in a safe place to discuss any problems

“*The more [the mothers] got to see the same person, the more information they tell you”* (Midwife Focus Group).

This may be particularly true for mothers who experienced intrusive thoughts and anxiety about whether a symptom was normal.

*“[The midwife] really helped relieve my over thinking brain”* (Mother 1).

“*There are some women that are always sort of worried about things that come up and wish they could just talk to someone … [The midwife] would have the chance to normalise a lot of what they’re experiencing*” (Doctor).

“*The lady has security in the sense that they’ve seen us before*” (Midwife 1).

Some participants indicated that continuity of care may be especially important in a mother’s first pregnancy.

### 3.2 Theme 2: Abandonment and the erosion of both caring and trusted relationships

Participants recommended extending continuity of care into the postpartum period due to the challenges mothers often face during this time.

*“I felt like the doctors and everyone brushed me off … when you’re pregnant they check up on you all the time, but then after you have the baby suddenly like no one cares anymore … but it was nice having the [continuity of care midwife]”* (Mother 1).

Some mothers described how the midwives that supported them on postnatal wards were unhelpful and how continuity of care from the same midwife would be helpful in this period.

“*I was still not getting that touch or connection from the nurses … being after this much trauma, I was expecting a bit more connection”* (Mother 9).

Mothers described wanting more support from midwives in the postpartum period, including someone to answer their questions and check in on them.

*“[A midwife] should call up after a few days, ask how you’re going and stuff … The first week or two weeks, I have questions, I don’t know who to ask”* (Mother 4).

Clinicians recommended that postpartum midwives contact antenatal continuity of care midwives to improve postpartum care for mothers with mental health concerns.

*“Certainly if there were postnatal patients with more significant mental health issues, it would be great to touch base with those continuity providers, and you’d hope that they would continue to provide that care”* (Doctor).

“One of the things we say is to make sure you talk about your birth afterwards and if there is something you do not understand to ask (Midwife Focus Group).

### 3.3 Theme 3: Mothers have different preferences regarding mode of contact from midwife

Mothers discussed preferring different types of communication from the continuity of care midwife, including face-to-face, video conference, phone, email and text messaging. Some mothers recommended face-to-face or video conference contact as it would make it easier for them to connect with the midwife and open up.

*“I loved the idea of email cause [the midwife] would email me whenever she was free. Then I’d get back to her when I was free … But the only other thing I could suggest is maybe doing a call or even like a video chat next time … Because when you’re talking sometimes it’s easier to let it out”* (Mother 2).

Not knowing the face of the midwife was challenging for some mothers when contact was only by phone or text messaging due to COVID-19.

*“I never actually met her. We only ever spoke over the phone or through text … she was still a stranger to me. I didn’t know who she was*” (Mother 1).

One participant shared how she felt bad contacting her midwife outside of business hours.

*“I’d feel bad to message [the midwife] at that time so I’d wait for business hours to message her”* (Mother 1).

### 3.4 Theme 4: Importance of a second opinion when providing continuity of care

Some participants highlighted how a second opinion from another clinician would be valuable in the midwife-led continuity of care model. Clinicians discussed how a drawback to the continuity of care model is the absence of a second opinion from another professional.

*“Sometimes it’s good to have a second pair of eyes to review the case because you might have missed something … you might have missed an important point because you see the patient week in, week out”* (Midwife 1).

One mother found

“with more midwives, [there is] more knowledge to have, more people get to see [her]. If one midwife doesn’t catch on to something, maybe another midwife will” (Mother 4).

*“For any situation where you have a single practitioner providing all the care, there is that small risk that that person is not competent or does not recognise the limits of their care and they might not refer on when needed”* (Doctor).

In cases where mothers needed to be supported by another clinician on the team, the continuity of care midwife could be a helpful source of knowledge and help mothers to engage with the care plan.

“*If we are providing care for a woman that has someone that’s been providing continuity of care, to be able to talk to that person is a good source of information as they know all aspects of care of that patient and up to date. It reduces the chances of us missing something or reduces chances of miscommunication … If you are worried about something, you can alert that person. They can keep an eye out for a certain issue or maybe if you’re planning an investigation and your patient might not understand fully, then you can relay to the [continuity of care midwife] and they might be able to encourage the [mothers] to participate in whatever care you’ve organised”* (Doctor).

## 4 Discussion

This study investigated the perspectives of mothers with mild-to-moderate mental health difficulties and clinicians regarding continuity of care during the perinatal period. Findings showed that mothers and clinicians described how continuity of care could positively affect a mother’s mental health by providing connection and rapport, information, and reassurance. According to participants, provision of rapport and information could improve effective and efficient care, such as helping mothers feel comfortable to disclose their symptoms and reducing unnecessary hospital presentations. Participants also had the perspective that continuity of care was important in the postpartum period. From the perspectives of mothers and clinicians, ability to access a second opinion in addition to continuity of care may be beneficial to ensure that the best care is provided.

This study showed that mothers perceive building rapport and trust with the same midwife during the perinatal period as positively impacting their mental health. In contrast, mothers who experienced a shared care model described distress in seeing different clinicians and needing to repeat their history. This is consistent with the findings from previous studies and systematic reviews (13, 19). Findings also support evidence that mothers experience increased connection and better treatment from seeing the same midwife (14, 29). Mothers who have a known midwife, sustained throughout their pregnancy are more likely to feel comfortable discussing their mental health concerns. However, if mothers do not trust their clinician or if they access multiple providers in a standard model of care, they can be reluctant to share their history and disclose perinatal mental health problems (19, 38, 39). Having mild-to-moderate depression in the perinatal period is related to risk factors including coming from a CALD background, experiencing childhood abuse, and experiencing physical or psychological intimate partner violence (10). These risk factors may make it even more difficult for mothers to disclose information to health professionals, indicating the importance of continuity of care for women with these backgrounds (40–42). Building rapport and having a relationship of trust with the continuity of care midwife may allow clinicians to provide accurate advice, interventions, and referrals, including helping mothers to access perinatal mental health services (27, 43). Interestingly, despite the study occurring in a multicultural setting and that several mothers were born overseas, culture or language were only discussed by one clinician who noted that increased rapport and disclosure occurred when a midwife could speak the same language as a mother. Further research about language concordance of service providers while serving culturally and linguistically diverse women would be beneficial (10, 29).

Our findings align with prior research that suggests mothers view midwives as a trustworthy source of information and that this information can ease women’s fears and provide reassurance (44, 45). While information provided by any midwife can be beneficial to mothers (45), continuity of care may offer mothers clarity regarding who they can contact if they have questions and may provide improved interventions by midwives due to their understanding of the patient’s history (46). Having access to the same midwife throughout the perinatal period may address mental health difficulties by putting women’s worries at ease (13). In addition, seeing the same patient repeatedly may assist midwives in detecting mental health difficulties and making appropriate referrals (29, 30). Mothers also value being able to access information flexibly, and at any time from the same midwife (47, 48). Emotional support provided by midwives is also experienced positively by women, such as listening to their experiences and providing reassurance that their pregnancies are progressing normally (48). In contrast, pregnant women can have negative experiences when their needs are not listened to by clinicians (48).

Mothers in this study highlighted the importance of receiving continued midwife support in the postpartum period, congruent with previous research (27, 49). Continuity of care may be particularly important for mothers with mild to moderate depression in pregnancy due to their increased risk of developing postpartum depression (10). Mothers may feel neglected during the postpartum period where professional support usually changes, decreases or stops (27, 50). Important physical and emotional needs are experienced by mothers following childbirth and professional follow-up care may assist in addressing these needs (50, 51). Providing continuity of care into the postpartum stage is associated with better mental health in mothers, better physical health in mothers and babies, and longer duration of breastfeeding (52). Continuity of care may also be particularly useful during crisis such as the COVID-19 pandemic which had an impact on postpartum care as it led to a reduction in postnatal appointments, partner attendance, no antenatal classes, and reduced staff numbers (53, 54). Some mothers in the study described poor experience with midwives on postnatal units, including not feeling connected. Previous research has shown that most mothers have positive experiences with staff on postnatal wards, although some experience the staff speaking in a ‘sharp’ manner or rate the care received on the postnatal ward as less positive compared with other stages of care (55, 56). Mothers may benefit from seeing the same midwife that was present during their pregnancy or birth on the postnatal ward following birth to help them feel more comfortable and facilitate the transition into the postpartum period (49).

The mode of communication with the midwife was also discussed by mothers, with some appreciating texting or emailing the midwife. This is relevant in this study as it was conducted during the COVID-19 pandemic (2021 period), and hence, during the numerous lockdowns and restrictions experienced in across New South Wales, Australia, seeing the continuity of care midwife face-to-face was not an option. Preferring texting communication is consistent with other research which shows that texting is an easy and efficient way for women to contact their midwife (57, 58). Other women in the study wanted to meet the midwife in person or via video to connect with them better (57). Therefore, it may be important for mothers to be provided with options of how they would like to communicate with their midwives to increase engagement. Furthermore, giving mothers the option to choose how to engage with the program midwives can aid in its implementation in maternity services. It would be beneficial in future research, however, to explore what can be done to support trust and rapport between mothers and clinicians in circumstances where midwife-led continuity of care is not possible or indeed not desirable.

According to the perspectives of participants in this study, midwife-led continuity of care may contribute to better use of hospital resources because mothers are reassured and able to disclose information to midwives, preventing unnecessary hospital presentations. This finding may partly explain prior evidence showing a cost-saving effect found in midwife-led continuity of care models (14, 59). Clinicians in this study also expressed the view that continuity of care may improve pregnancy and birth outcomes, consistent with previous quantitative research (14). Continuity of care throughout pregnancy may also improve women’s experiences of labour and birth, which are related to a decreased risk of mental health problems (60–62). When mothers are being cared for by one clinician during the perinatal period, participants in this study discussed the importance of a second opinion from another health professional. In the Australian context, mothers with higher risk pregnancies are referred to medical and allied health appointments, having a primary midwife working collaboratively with other clinicians in the mother’s care has proven to be advantageous. Seeing multiple midwives or clinicians may improve support for mothers by providing a range of expertise and reducing complacency (63, 64). Mothers who desire a second opinion may benefit from a midwifery group practice where continuity of care is provided by a small group of midwives.

### 4.1 Strengths and limitations

A strength of this research is the focus on mothers with EPDS scores of 10 to 12 (which is just below the clinical cut off for anxiety and depression) who are often excluded from clinical programs as they are deemed not severe enough to meet the intake criteria for formal psychiatric services. As a result, they are also often not included in research regarding perinatal mental health difficulties, despite the increased risk for postpartum depression. In addition, this study provides novel perspectives from mothers in an ethnically diverse and socioeconomically disadvantaged area regarding the positive nature of having a continuity of care midwife. This study also had several limitations. First, participants received various models of care as part of their ‘routine care’, which may have impacted their perspectives and access to midwifery continuity of care. Second, fathers/partners were not included in the study, it therefore remains unclear what impact midwifery continuity of care has on the family as a whole. Third, the was a small and unequal representation of health professionals who participated in the study which may reduce the generalisability of results. Fourth, interview data and focus group data were analysed together. While comparing focus group and interview data can provide valuable insights it also presents several limitations. Some of these limitations include (1) group versus individual dynamics, with group interactions influencing responses due to social dynamics; (2) different level of detail elicited, with focus groups providing less detailed data than individual interviews; and (3) overgeneralisation of findings from focus groups to individual participants. Future research would benefit from using one method for data collection.

### 4.2 Future research and practice implications

Future research would benefit from investigating whether midwife-led continuity of care can prevent postpartum depression in mothers with low to moderate depression in pregnancy. More research is also needed to determine how midwife-led continuity of care affects hospital resources and efficiency of care, such as whether hospital presentations are reduced.

This research extends previous research by highlighting the importance of offering mothers with mild to moderate depression midwife-led continuity of care during the perinatal period (13) as the attitudes of this group are reflective of women with more severe concerns. It is important that continuity of care midwives are trained in how to care for mothers with mental health challenges and understand resources that they can provide to these mothers (65). Midwives practicing in continuity of care models may benefit from peer consultations and reflective practice to ensure that they are providing the best care to their patients (66) and also for themselves.

## Data Availability

The raw data supporting the conclusions of this article will be made available by the authors, without undue reservation.
