# Supplementary material for: Perinatal continuity of care for mothers with depressive symptoms: perspectives of mothers and clinicians
Source: Front Psychiatry. 2024 Sep 19;15:1385120. doi: 10.3389/fpsyt.2024.1385120 (PMC11447617; doi:10.3389/fpsyt.2024.1385120)
Supplement: Supplementary file 1 [file DataSheet1.docx]

Appendix 1

**Interview questions for mothers**

1. What do you remember about the midwife that you saw as a result of participating in the research project?

2. What sorts of things or topics do you remember discussing with the midwife?

3. What did it feel like to have access to the midwife?

4. Do you think that having access to the midwife effected your mental health in any way? If so, how?

5. Do you think having access to this program would benefit others? Why/why not?

6. Was there anything that surprised you as a result of participating in this program? If yes, what was it? Why? If no, why not?

7. Is this your first pregnancy? If not, how did the care you receive as a result of participating in this program differ from the care you received previously?

8. Did your participation in this program cause you to do anything that you wouldn’t have done otherwise? Or avoid doing anything? Or think about anything new?

9. Was the programhelpful or unhelpful for you at the time? Why or why not?

10. Now that your baby is XX months, with the benefit of hindsight, do you think that the program was helpful or unhelpful for you? In what ways?

11. If you had a friend who was considering whether or not to participate in the program, what would you say to them?

12. If the research team wanted to improve the program and they asked for your suggestions, what would you recommend?

**Interview questions for clinicians**

1. What do you remember about this research project, do you remember what were we trying to do? (Note if the health professional doesn’t remember it is okay to remind them what the study is about and provide a brief outline).

2. What is your role within the health system, and how would someone in this position be impacted by this study?

3. Did the women in your care discuss the study? What was their impression of the study and the support provided?

4. Was it disruptive to manage clients who were in different study conditions? (It was probably business as usual for most health staff).

5. Were you able to tell which clients had access to the midwife and which did not? Why/why not?

6. Do you think that having access to the midwife had an impact on clients’ mental health? Why/Why not?

7. Do you think having access to this program would benefit others? Why/why not?

8. Were you largely unaware whether the women in your care participated in the study? Would it have been useful for you to know this information?

9. Can you think of any benefits related to having a midwife that is in touch with you throughout the perinatal period?

10. Can you think of any risks related to having a midwife that is in touch with you throughout the perinatal period?

11. Did your participation in this program cause you to do anything that you wouldn’t have done otherwise? Or avoid doing anything? Or think about anything new?

12. If the research team wanted to improve the program and they asked for your suggestions, what would you recommend?
